# Association analysis of the glucocorticoid receptor gene (*NR3C1*) haplotypes (ER22/23EK, N363S, BclI) with mood and anxiety disorders in patients with asthma

**DOI:** 10.3892/etm.2014.1734

**Published:** 2014-05-28

**Authors:** MICHAŁ PANEK, TADEUSZ PIETRAS, JANUSZ SZEMRAJ, PIOTR KUNA

**Affiliations:** 1 Department of Internal Medicine, Asthma and Allergy, Medical University of Łódź, Łódź, Łódź Voivodeship 90-153, Poland; 2 Department of Pneumology and Allergology, Medical University of Łódź, Łódź, Łódź Voivodeship 90-153, Poland; 3 Department of Medical Biochemistry, Medical University of Łódź, Łódź, Łódź Voivodeship 90-153, Poland

**Keywords:** glucocorticoid receptor gene *NR3C1*, inflammation, asthma, depression, anxiety, breathlessness

## Abstract

Chronic inflammation in the bronchi of long-term asthma patients worsens mood disorders, which has been shown to correlate with elevated levels of multiple proinflammatory cytokines. The glucocorticoid receptor (GR) gene, *NR3C1,* plays a key role in the control of inflammation. Disturbances in the structure and function of the GR alter the glucocorticoid regulation of the corticotropin-releasing hormone, which leads to nonspecific activation of numerous receptors in the brain and alters the metabolism. The aim of the present study was to evaluate the role of *NR3C1* haplotypes in mood and anxiety disorders. The study included 235 patients with asthma and 216 healthy individuals. Genotyping of *NR3C1* gene polymorphisms was performed using polymerase chain reaction-restriction fragment length polymorphism. Beck’s Depression Inventory, State and Trait Anxiety Inventory tests and the Borg scale were applied for all the subjects. Significant differences in the levels of depression (P=0.000008) and dyspnea (P=0.000001) were observed between the patients and healthy subjects. In addition, a correlation was identified between spirometric parameters and the intensity of depression, anxiety and subjective dyspnea. The AA ER22/23EK, AA N363S and CC BclI haplotype of the *NR3C1* gene was identified to significantly aggravate trait anxiety in patients with asthma (P=0.026). Therefore, the *NR3C1* gene substantially modified the level of trait anxiety in asthma sufferers.

## Introduction

Affective disorders and obstructive diseases, including asthma and chronic obstructive pulmonary disease (COPD), are becoming more frequent (1). The growth in the number of asthma sufferers has increased with the incidence of mood disorders, including asthma and mood disorder, both, dependent on genetic and environmental factors. There is a significant prevalence of depression in daily life with 7–12% of males and 20–25% of females affected, and an annual occurrence of 6–12% in the adult population. Females are two or three times more likely to suffer from depression compared with males, and the probability of depression recurring after treatment during a lifetime is as high as 80% (1). The relative occurrence of mood disorders in the course of obstructive disease is 1.7 times greater in asthma sufferers [95% confidence interval (CI), 1.1–2.3] and 1.9 times greater in patients with COPD (95% CI, 1.2–2.1) (2). In the stable phase of COPD, depression affects between 15 and 42% of patients, while mood disorders affect 10–19% (3). Mild depression is estimated to occur in 26% of mild asthma sufferers and moderate symptoms of depression may be present in 36% of individuals with moderate asthma (4).

Depression and anxiety, as components of personality, are to a certain degree conditioned by temperament, which is a natural predisposition of human emotional reactivity. Temperament is manifested in the formal elements of behavior; the reaction of a person to stress and their behavior in extreme situations depends indirectly on their temperament. Certain elements of temperament predispose the patient to the development of disorders in their psyche, including depression and anxiety, and behavior (5–7).

Psychosomatic factors play a significant role in the pathogenesis of asthma (8). Mood and anxiety disorders are more common in patients with obstructive disorders compared with healthy control groups. Stress, such as that encountered when not carrying an inhaler, may cause psychogenic dyspnea, which is demonstrated by a positive association existing between the functional parameters of the flow-volume curve and the subjective sense of dyspnea and anxiety (4).

There are several important mechanisms that induce mood disorders in asthma, including common genetic factors, certain drugs used to treat obstruction and inflammatory mediators (cytokines) that modify the metabolism of the brain. Moreover, asthma as a chronic inflammatory airway disease acts as a stressor (4,9–13).

Asthma is a chronic inflammatory disease of the respiratory tract that exacerbates mood disorders and is correlated with the concentration of a number of inflammatory markers, including C-reactive protein, interleukin (IL)-5, -6 and -12, tumor necrosis factor-α and interferon-α (10–12). Inflammatory mediators cause a secondary decrease in the activity of the cAMP response element-binding and tyrosine kinase transforming proteins, resulting in impaired secretion of brain-derived neurotrophic factor in the frontal lobes and limbic system (12,14–16). As a consequence of these reactions, the hippocampus becomes damaged and a there is a reduced concentration of monoamines in the brain (14,17). Certain cytokines are able to activate multiple signaling pathways with the activation of janus kinase (JAK) 2 and STAT5. The JAK-STAT signaling pathway plays an important role in cell proliferation and survival in the central nervous system (CNS). The pathway also affects the cell response to various hormones, growth factors and cytokines (18,19). Activation of the JAK-STAT pathway leads to the development of depressive effects via glucocorticoid (GC) signaling (20).

Hyperactivity of the well characterized hypothalamic-pituitary-adrenal (HPA) axis, plays an important role in the pathogenesis of affective disorders in asthma. In addition, corticotrophin-releasing hormone (CRH) hyperactivity causes disorders in the proper functioning of the CNS, due to impaired negative regulation of CRH by GCs (21–23). This altered regulation of CRH by GCs is caused by disturbances to the structure and function of the GC receptor (GR) (23,24). Numerous studies have demonstrated the crucial role played by polymorphic forms of the gene coding for the GR, *NR3C1,* in the regulation of CRH (25–29). Changes in the nucleotide sequence by single nucleotide polymorphisms (SNPs) may influence expression and lead to changes in RNA assembly. Polymorphisms are responsible for the modification of the secondary and tertiary structure of GR domains, and cause disorders in the initiation and stability of mRNA transcription for GRs (25,26,28,30,31). The BclI (rs41423247) and N363S (rs6195) polymorphisms of the *NR3C1* gene increase the sensitivity of the GCs, while the ER22/23EK SNP (rs6189/rs6190) is associated with resistance to GCs (28,32,33).

Therefore, the aims of the present study were twofold: Firstly, to determine whether correlations exist between the levels of depression and anxiety and more objective measures of airflow obstruction in asthma patients, and secondly, to confirm whether the genetic determinant of *NR3C1* significantly affects these factors.

## Patients and methods

### Ethical approval

The study was approved by the local Ethics Committee (Consent of Research Review Board of the Medical University of Łódź, Łódź, Poland; no. RNN/133/09/KE). At the start of the study, participants were invited to attend voluntarily and prior to enrollment, written informed consent was obtained from every patient.

### Patient selection

A total of 235 patients with bronchial asthma were recruited for the study. Asthma diagnosis was established according to the Global Initiative For Asthma recommendations, based on clinical asthma symptoms and lung function tests. The level of asthma severity and control was determined according to the American Thoracic Society (ATS) guidelines (34). Apart from a subjective examination, structured anamnesis was performed and a number of factors were examined, including gender, obesity, tobacco smoking, duration of bronchial asthma, allergy to house dust mites, animal fur, mould spores, cockroach allergens and hypersensitivity to nonsteroidal anti-inflammatory drugs (25,26).

The exclusion criteria included subjects suffering from clinically significant exacerbations, or who were using drugs, such as rifampicin, phenobarbital, phenytoin or ephedrine, which may induce resistance to GCs. Subjects with signs of viral infections, generalized or affecting the respiratory tract, as well as those failing to comply with the recommendations of their doctor, were also excluded. The control arm included a group of 216 healthy adults who met the following criteria: No history or symptoms of bronchial asthma, other pulmonary diseases, allergy, atopic dermatitis and hypersensitivity to aspirin; negative skin tests results for 12 common allergens; and no first-degree relatives with bronchial asthma or atopic disorders. Healthy volunteers were selected on a random basis from the general population (25,26).

In the bronchial asthma group, 62.6% (147) of the patients were female and 37.4% (88) were male. The average age was 48.8±16.0 years (range, 19–82 years; median, 51 years; mode, 52 years). The average forced expiratory volume in 1 sec (FEV1) was 2.2±0.9 litres (72.7%; median, 2.2 litres; mode, 2.4 litres) and the average forced vital capacity (FVC) was 3.3±1.1 litres (91.4%; median, 3.2 litres; mode, 2.3 litres).

The control group comprised 216 healthy individuals: 65.7% (142) females and 34.3% (74) males. The average age was 45.7±16.3 years (range, 18–85 years; median, 47 years; mode, 23 years). The average FEV1 was 3.0±0.8 litres (96.1%; median, 2.9 litres; mode, 2.7 litres) and the average FVC was 3.8±1.0 litres (102.7%; median, 3.6 litres; mode, 3.5 litres).

Detailed descriptive statistics for age and spirometric parameters for the cases and controls are presented in Table I.

### Functional assessments

Functional assessments of the respiratory system were conducted according to the European Respiratory Society and ATS standards (34).

In all the tests, the Polish language version of the Beck Depression Inventory was used (35–37), where results were expressed as a total number of the points obtained. Trait and state anxieties were measured using the Polish language adaptation of Spielberg’s State and Trait Anxiety Inventory (STAI) (37,38), where the results were expressed as absolute numbers of the points obtained. The level of declared breathlessness was estimated on the 10-point Borg scale of subjective feelings (37,39). Each patient recorded their subjective impression of breathlessness at the time of the test on a scale of 0–10 (37).

### Sample collection

Venous blood samples were collected from the participants and placed into K3-EDTA tubes. DNA was obtained from the peripheral blood leukocyte fraction and isolated using a QIAamp DNA Blood Mini kit (Qiagen, Hilden, Germany), according to the manufacturer’s instructions. Polymorphisms were analyzed using polymerase chain reaction-restriction fragment length polymorphism (PCR-RFLP).

### PCR

Exponential amplification of the DNA segments for the ER22/23EK polymorphism was conducted using forward (5′-TGC ATT CGG AGT TAA CTA AAA AG-3′) and reverse (5′-ATC CCA GGT CAT TTC CCA TC-3′) primers, according to a standard PCR protocol. Starter binding to complementary DNA matrix sites was conducted at 56°C. Amplified DNA sequences of 448 bp were obtained. The genetic material was incubated with the *Mnl*I restriction enzyme (Fermentas International, Inc., Burlington, Canada) at 37°C for 20 h (40). DNA fragments of 149 and 163 bp, and shorter fragments containing 50, 49 and 35 bp, were obtained as a set of representative, typical (wild type) alleles, whereas segments of 163 and 184 bp, and shorter fragments containing 50 and 49 bp, constituted the set of polymorphic alleles (26,40).

Exponential amplification of the DNA segments for the N363S polymorphism was performed using forward (5′-CCA GTA ATG TAA CAC TGC CCC-3′) and reverse (5′-TTC GAC CAG GGG AAG TTC AGA-3′) primers, according to a standard PCR protocol (41). Starter binding to complementary DNA matrix sites was conducted at 56°C and amplified DNA sequences of 357 bp were obtained. The material was incubated with FastDigest^®^ *Tsp509*I (*Tas*I) restriction enzyme (Fermentas International, Inc., Burlington, Canada) at 65°C for 1 h (40). DNA fragments of 135, 73, 70, 60 and 19 bp were obtained as a set of representative, typical (wild type) alleles, whereas segments of 135, 92 (73 and 19 bp), 70 and 60 bp constituted the set of polymorphic alleles (25,41).

Amplification of the DNA fragment containing the BclI polymorphism of the *NR3C1* gene was conducted using starters with the following sequences: Forward, 5′-GAG AAA TTC ACC CCT ACC AAC-3′ and reverse (5′-AGA GCC CTA TTC TTC AAA CTG-3′, according to a standard PCR protocol (42). Starter binding to complementary DNA matrix sites was conducted at 56°C. The *Bcl*I restriction enzyme (Fermentas International, Inc.) was used for the digestion of the amplification product containing the *Bc1*I polymorphism (40). Hydrolysis of the PCR product with the restriction enzyme was conducted for 24 h at 55°C. DNA fragments containing 263 and 151 bp were identified as a set of representative, typical (wild type) alleles, as well as segments with 418, 263 and 155 bp. An RFLP product, 418 bp in length, was identified as a set of polymorphic alleles (40).

For each of the SNP tests, representative, typical homozygotes and heterozygotes were sequenced and used as internal controls. Following restriction enzyme digestion, 4 ml indicator dye was added to the test tube. Electrophoresis was performed using an 8% polyacrylamide gel with 1:20 Tris-acetate-EDTA buffer at 120 V for 60 min. The gel was stained with 0.5 mmol/ml ethidium bromide and imaged under ultraviolet light using a camera and Image Master software (Pharmacia Biotech, Tokyo, Japan). Electropherograms of the amplified products following restriction enzyme digestion were photographed and saved on digital media. Images were analyzed using Image Master software.

### Statistical analysis

Statistical analysis was performed using univariate analysis of variance. An advanced regression model (general linear model) was used to evaluate the dependencies between multiple variables. Statistical analysis was performed using STATISTICA data analysis software system, version 10 (AXAP202E504303AR-A; StatSoft, Inc., Tulsa, OK, USA). In addition, the Bonferroni correction was used for the three tested polymorphisms. P<0.05 was considered to indicate a statistically significant difference. Differences and linear trends between the three tested genotypes were identified. A haplotype effect model (additive and dominant) was used to analyze the haplotypes and haplotype-specific scores with the R statistics package (http://www.r-project.org/). Genotyping was performed by two investigators who were unaware of the phenotypes.

## Results

In the test and control groups, the following values were identified for the studied variables: Beck (depression), STAI-I (state anxiety), STAI–II (trait anxiety) and Borg (breathlessness) scales, as presented in Table II.

The frequencies of occurrence of the polymorphic forms of *NR3C1* are presented in Table III. The AA ER22/23EK, GG N363S and CC BclI alleles were identified to be rare forms of *NR3C1* polymorphisms, while GG ER22/23EK, AA N363S and GG BclI were more frequent. Univariate analysis of the tested parameters revealed the existence of significant differences between the test and control groups, as shown in Table IV.

In the studied populations, a complex association was identified between the analyzed variables (depression, state and trait anxieties and breathlessness) and the results of the respiratory function tests. Table V presents detailed correlations divided into the two subgroups: Healthy subjects and asthma patients.

### Functional assessments

Associations between the variables (depression, anxiety and breathlessness) and objective, measurable and repeatable spirometric parameters were analyzed. These results illustrated the degree of airway obstruction in the asthma patients with regard to the severity of the illness, as assessed by the ATS criteria, compared with the control group. A detailed analysis is presented in Table VI.

### Haplotype analysis

The results of more advanced tests concerning the polymorphisms of the *NR3C1* gene, which is located on the long arm of chromosome 5 at position q31–q32, are shown in Tables VII–X (42). The gene is inherited as a set of associated alleles, together with depression, anxiety and breathlessness. Haplotype analysis performed using the additive haplotype effects model [global statistic, 0.913; degrees of freedom (DF), 6; P=0.988] did not reveal any correlations between the *NR3C1* gene haplotypes and depression, as shown in Table VII. In addition, haplotype analysis performed using the additive haplotype effects model (global statistic, 1.237; DF, 6; P=0.974) revealed that no combinations of alleles were significantly associated with state-anxiety (Table VIII). However, haplotype analysis using the haplotype-specific stores did confirm a statistically significant association (P=0.026) with trait anxiety for one of the combinations of alleles, as shown in Table IX. Haplotype analysis performed using the additive haplotype effects model (global statistic, 6.195; DF, 6; P=0.401) did not reveal an association between *NR3C1* haplotypes and breathlessness, as shown in Table X. Therefore, haplotype analysis revealed a correlation between polymorphic forms of *NR3C1* and the level of trait-anxiety.

### Correlation analysis

**Figure 1:**
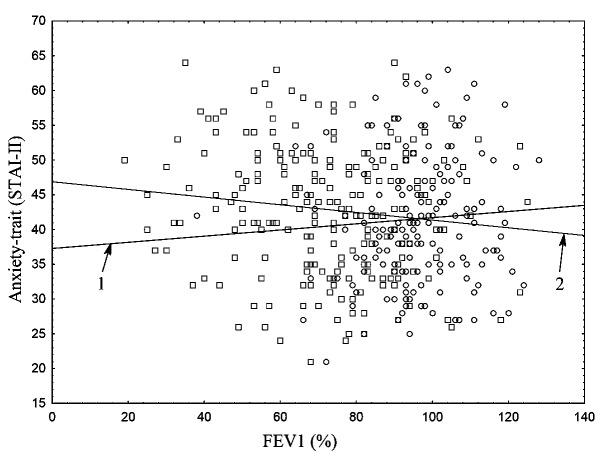
Correlation between STAI–II and FEV1 (%) for the cases and controls. Line 1 (circles), healthy controls; line 2 (squares), asthma patients; FEV1, forced expiratory volume in 1 sec; STAI–II, trait-anxiety inventory.

**Figure 2:**
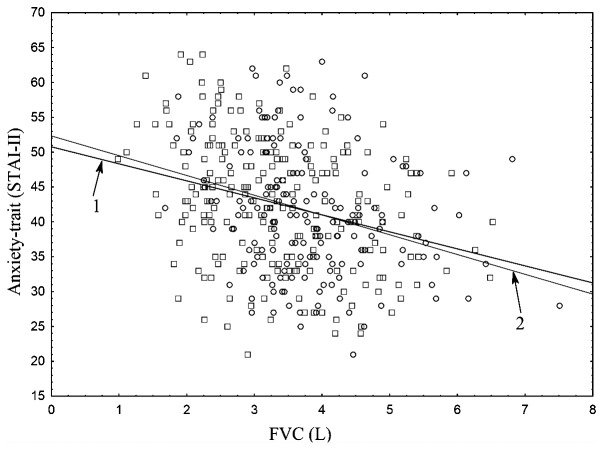
Correlation between STAI–II and FVC for the cases and controls. Line 1 (circles), healthy controls; line 2 (squares), asthma patients; FVC, forced vital capacity; STAI–II, trait-anxiety inventory.

Scatter plots (Figs. 1 and 2) presented a graphical interpretation of the key correlations between trait-anxiety (STAI–II) and the spirometric parameters, FEV1 and FVC, for the following groups: Cases vs. controls.

## Discussion

Research on the interaction between GCs and receptors is important for improving the understanding of asthma therapy. The use of GCs was a turning point in the history of the treatment of this disease. The *NR3C1* gene and its transcripts are key factors involved in the inflammation of asthma and are responsible for alterations in the functioning of the GR (22–24). GCs are able to alter the regulation of CRH, thus, affect the development of depression and anxiety disorders (22–24). Therefore, polymorphic forms of *NR3C1,* which replicate the effect of the regulatory gene and affect the function of the promoter and the encoded protein (GR), can lead to the induction of depressive and anxiety disorders, and modify their intensity.

In the present study, a statistically significant correlation was observed between the levels of depression, anxiety (state and trait) and dyspnea with the spirometric parameters FEV1 and FVC. In addition, a correlation was observed between the FEV1:FVC respiratory function with depression and shortness of breath. These associations were observed in the asthma patients and control group.

Advanced analysis concerning the severity of asthma was performed by dividing the group of patients into two subgroups based on the ATS criteria. A correlation was observed between the level of depression in patients with severe asthma refractory to treatment and FEV1 (r=−0.227, P=0.048) and between the level of trait anxiety and FVC (r=−0.321, P=0.004). Similar observations were identified in asthma patients who did not meet the criteria of severe asthma refractory to treatment (depression vs. FEV1; depression vs. FVC; state-anxiety vs. FVC; state-anxiety vs. FEV1:FVC; trait-anxiety vs. FEV1, trait-anxiety vs. FVC; dyspnea vs. FEV1; dyspnea vs. FEV1; dyspnea vs. FVC; depression vs. FEV1:FVC).

A total of 24 *NR3C1* gene variants were detected for each of the tested variables (depression, state and trait anxiety and breathlessness). Only one of the *NR3C1* haplotypes exhibited a correlation with state-anxiety (P=0.026), which was AA ER22/23EK (rare), AA N363S (common) and CC BclI (rare).

The ER22/23EK polymorphism is composed of two transition nucleotides at the 22^nd^ and 23^rd^ codons, which are connected with each other. SNPs may alter the secondary structure of the GR mRNA, and may consequently initiate translation from the 1^st^ or the 27^th^ methionine, as well as affect the stability of the mRNA (26,32,33,43,44).

The presence of the N363S polymorphism promotes structural changes in the A/B region of the GR, affecting activation function-1 domain, which interacts with multiple transcription factors, as well as within the activator protein-1 functional domain (25,26,45). The N363S polymorphism modulates numerous regulatory protein groups, decreases the activity of nuclear factor-κB and stimulates the production of IκBα, thus, interfering with the suppression of IL-2 (25,26,45).

The BclI SNP is coupled with two other polymorphic forms of *NR3C1*: Intron B 33389 (rs33389) and Intron B 33388 (rs33388) (27,46). These three polymorphisms modify the recognition site by alternative splicing of the *NR3C1* factors: SR (serine/arginine-rich protein) and SF2/ASF (splicing factor 2/alternative splicing factor) (27,47).

The results of the present study confirm previous observations that changes, including depression, anxiety and breathlessness, significantly correlate with spirometric parameters and have an influence on the severity of the course of asthma (4,37). The present study characterizes and precisely describes the haplotypes of the *NR3C1* gene, while identifying the haplotype responsible for the changes occurring in trait-anxiety in asthma sufferers. The results demonstrated that trait-anxiety significantly lowered a number of spirometric parameters (FEV1, FVC, FEV1:FVC), causing a more severe course of illness. Previous studies on *NR3C1* gene polymorphisms have failed to confirm that the ER22/23EK and BclI SNPs play any role in the etiopathogenesis of asthma. The results of the current study confirmed that all *NR3C1* polymorphisms (haplotype, ER22/23EK, N363S and BclI) participate in the regulation of the intensity of trait-anxiety in asthma patients, which significantly correlates with increases in airflow obstruction (4,25,26,37,48). The association between *NR3C1* haplotypes with depression and anxiety may therefore be complex. The same polymorphism which influences the development of asthma and the degree of severity can function indirectly as a stressor (airway obstruction), inducing depression and anxiety disorders. This observed phenomenon may also explain the differences in the functioning of the GR associated with the decreased expression of multiple proteins in the brain. Co-occurrence of asthma, mood disorders and anxiety may stem from a common genetic cause; this has been indirectly confirmed by the evidence that gene polymorphisms are associated with the occurrence of *NR3C1* mood disorders, and also that the HPA axis is involved in the pathogenesis of depression.

Therefore, the gene encoding *NR3C1* GR activity is an important regulator of the biochemical and molecular mechanisms involved in the changes of the GR, thus, affects the degree of airway obstruction. The haplotype, ER22/23EK, N363S and BclI, materially affects, directly or indirectly, psychopathological and personality variables, including depression, anxiety and shortness of breath (4,25,26,37,48).

Furthermore, the present study highlights the under diagnosis of affective and anxiety disorders among asthma sufferers.

However, the present study has limitations associated with asthma medication treatment. This group of medicines comprises inhaled and systematic glucocorticosteroids, which can induce depression. Therefore, patients experiencing asthma attacks, which required the use of glucocorticosteroids, were excluded from the study. In addition, the test and the control group did not include subjects who were receiving long-term systematic glucocorticosteroid treatment for any other medical conditions.

For the subjects included in the study, the diagnosis of depression and anxiety disorders was performed for the first time. Thus, it was difficult to further assess the clinical course of the affective and anxiety disorders. Patients who had previously been treated for anxiety disorders and depression were not included in the study.

In conclusion, a multivariate study analyzing the role of genetic variants in the *NR3C1* gene was performed in patients with asthma, with particular emphasis on the intensity of their depression, anxiety and shortness of breath.

The results demonstrated that the haplotypic variations in the regulatory regions of the *NR3C1* gene significantly correlated with trait-anxiety. In addition, important molecular mechanisms leading to the development of depression and anxiety in asthma patients were identified, and correlations that existed with functional spirometry parameters were indicated.

*NR3C1* polymorphisms and haplotypes are general modulating factors of the level of depression, shortness of breath and anxiety, which significantly predispose patients with asthma to the development of affective disorders and anxiety.

## Figures and Tables

**Table I:** Descriptive statistics for age and spirometric parameters in the healthy control subjects and asthma patients.

Parameter	Bronchial asthma group	Control group
Subjects (n)	235	216
Females (%)	62.6	65.7
Males (%)	37.4	34.3
Age (years)		
Average	48.8	45.7
SD	±16.0	±16.3
Minimum	19	18
Maximum	82	85
Median	51.0	47.0
Mode	52.0	23.0
FEV1		
Average (litre)	2.2	3.0
Average (%)	72.7	96.1
SD (litre)	±0.9	± 0.8
Median (litre)	2.2	2.9
Mode (litre)	2.4	2.7
FVC		
Average (litre)	3.3	3.8
Average (%)	91.4	102.7
SD (litre)	±1.1	±1.0
Median (litre)	3.2	3.6
Mode (litre)	2.3	3.5

SD, standard deviation; FEV1, forced expiratory volume in 1 sec; FVC, forced vital capacity.

**Table II:** Descriptive statistics of depression, anxiety and breathlessness in the healthy control and asthma patients.

A, Controls						

Parameter	Mean	Median	Mode	Min.	Max.	SD
Beck scale	8.34	6.00	2.00	0.00	33.00	7.14
STAI–I	36.95	35.00	1.00	20.00	62.00	9.29
STAI–II	41.54	41.00	1.00	21.00	63.00	9.06
Borg scale	1.74	1.00	0.00	0.00	8.00	1.89

B, Cases						

Parameter	Mean	Median	Mode	Min.	Max.	SD

Beck scale	10.28	9.00	1.00	0.00	45.00	7.65
STAI–I	38.19	37.00	1.00	20.00	74.00	10.60
STAI–II	42.91	43.00	1.00	1.00	64.00	9.17
Borg scale	3.48	3.00	5.00	0.00	9.00	2.48

STAI-I, state anxiety inventory; STAI–II, trait anxiety inventory; SD, standard deviation.

**Table III:** Frequencies of polymorphic forms of *NR3C1* in the healthy controls and asthma patients.

*NR3C1* polymorphic form	Controls (%)	Cases (%)
ER22/23EK G	96.74	97.22
ER22/23EK A	3.26	2.78
N363S A	86.74	87.45
N363S G	13.26	12.55
BclI G	58.80	57.05
BclI C	41.20	42.95

**Table IV:** Associations between the variables in the test and control groups using univariate analysis of variance.

Variable	SS	DF	MS	F	P-value
Beck scale	1285.840	2	642.920	12.107	<0.001
STAI-I	307.600	2	153.800	1.526	0.218
STAI–II	443.500	2	221.700	2.676	0.070
Borg scale	387.139	2	193.569	40.220	<0.001

An overall analysis was performed for the two groups together. SS, sum of squares; DF, degrees of freedom; MS, mean square; F = MSM/MSE; MSM, mean square model; MSE, mean square error; STA-I, state anxiety inventory; STA-II, trait anxiety inventory.

**Table V:** Advanced multivariate general regression models for depression, anxiety and breathlessness in the group of asthma patients compared with the healthy control group.

A, Depression (Beck scale)		

Spirometric parameter	Healthy subjects	Asthma patients
FEV1 (litre)	r=−0.2544	r=−0.3759
	P=0.0007	P<0.0001
	r^2^=0.0647	r^2^=0.1413
FVC (litre)	r=−0.2300	r=−0.3495
	P=0.0023	P<0.0001
	r^2^=0.0529	r^2^=0.1221
FEV1:FVC (%)	r=−0.1069	r=−0.1876
	P=0.1615	P=0.0052
	r^2^=0.0114	r^2^=0.0352

B, State-anxiety (STAI-I)		

Spirometric parameter	Healthy subjects	Asthma patients

FEV1 (litre)	r=−0.1430	r=−0.1517
	P=0.0605	P=0.0244
	r^2^=0.0205	r^2^=0.0230
FVC (litre)	r=−0.1488	r=−0.2217
	P=0.0507	P=0.0009
	r^2^=0.0222	r^2^=0.0491
FEV1:FVC (%)	r=−0.0147	r=0.0893
	P=0.8482	P=0.1867
	r^2^=0.0002	r^2^=0.0080

C, Trait-anxiety (STAI–II)		

Spirometric parameter	Healthy subjects	Asthma patients

FEV1 (litre)	r=−0.2651	r=−0.2810
	P=0.0004	P<0.0001
	r^2^=0.0703	r^2^=0.0789
FVC (litre)	r=−0.2618	r=−0.3397
	P=0.0005	P<0.0001
	r^2^=0.0686	r^2^=0.1154
FEV1:FVC (%)	r=−0.0500	r=0.0248
	P=0.5133	P=0.7142
	r^2^=0.0025	r^2^=0.0006

D, Breathlessness (Borg scale)		

Spirometric parameter	Healthy subjects	Asthma patients

FEV1 (litre)	r=−0.1948	r=−0.3202
	P=0.0107	P<0.0001
	r^2^=0.0379	r^2^=0.1025

Spirometric parameter	Healthy subjects	Asthma sufferers

FVC (litre)	r=−0.1506	r=−0.3048
	P=0.0493	P<0.0001
	r^2^=0.0227	r^2^=0.0929
FEV1:FVC (%)	r=−0.2194	r=−0.1512
	P=0.0039	P=0.0249
	r^2^=0.0481	r^2^=0.0229

FEV1, forced expiratory volume in 1 sec; FVC, forced vital capacity; FEV1:FVC, ratio of FEV1 to FVC; r, linear correlation coefficient; r^2^, multiple correlation coefficient squared; P, P-value.

**Table VI:** Advanced multivariate general regression models for depression, anxiety and breathlessness in the asthma patient group compared with the control group, with regard to the severity of asthma.

A, Depression (Beck scale)			

Spirometric parameter	Healthy	ATS 0	ATS 1
FEV1 (litre)	r=−0.2544	r=−0.3630	r=−0.2273
	P=0.0007	P<0.0001	P=0.0483
	r^2^=0.0647	r^2^=0.1318	r^2^=0.0517
FVC (litre)	r=−0.2300	r=−0.3687	r=−0.2099
	P=0.0023	P<0.0001	P=0.0688
	r^2^=0.0529	r^2^=0.1360	r^2^=0.0441
FEV1:FVC (%)	r=−0.1069	r=−0.0686	r=−0.1422
	P=0.1615	P=0.4139	P=0.2204
	r^2^=0.0114	r^2^=0.0047	r^2^=0.0202

B, State anxiety (STAI-I)			

Spirometric parameter	Healthy	ATS 0	ATS 1

FEV1 (litre)	r=−0.1430	r=−0.1552	r=−0.0805
	P=0.0605	P=0.0633	P=0.4895
	r^2^=0.0205	r^2^=0.0241	r^2^=0.0065
FVC (litre)	r=−0.1488	r=−0.2183	r=−0.1899
	P=0.0507	P=0.0086	P=0.1003
	r^2^=0.0222	r^2^=0.0476	r^2^=0.0361
FEV1:FVC (%)	r=0.0767	r=0.1753	r=0.1775
	P=0.3156	P=0.0356	P=0.1250
	r^2^=0.0059	r^2^=0.0307	r^2^=0.0315

C, Trait anxiety (STAI–II)			

Spirometric parameter	Healthy	ATS 0	ATS 1

FEV1 (litre)	r=−0.2651	r=−0.2724	r=−0.2186
	P=0.0004	P=0.0010	P=0.0578
	r^2^=0.0703	r^2^=0.0742	r^2^=0.0478
FVC (litre)	r=−0.2618	r=−0.3221	r=−0.3210
	P=0.0005	P<0.0001	P=0.0047
	r^2^=0.0686	r^2^=0.1038	r^2^=0.1031
FEV1:FVC (%)	r=0.0519	r=0.1093	r=0.1400
	P=0.4977	P=0.1923	P=0.2276
	r^2^=0.0027	r^2^=0.0119	r^2^=0.0196

D, Breathlessness (Borg scale)			

Spirometric parameter	Healthy	ATS 0	ATS 1

FEV1 (litre)	r=−0.1948	r=−0.3043	r=−0.1254
	P=0.0107	P=0.0002	P=0.2773
	r^2^=0.0379	r^2^=0.0926	r^2^=0.0157
FVC (litre)	r=−0.1506	r=−0.2907	r=−0.1909
	P=0.0493	P=0.0004	P=0.0963
	r^2^=0.0227	r^2^=0.0845	r^2^=0.0365
FEV1:FVC (%)	r=−0.2194	r=−0.1256	r=0.0500
	P=0.0039	P=0.1350	P=0.6656
	r^2^=0.0481	r^2^=0.0158	r^2^=0.0025

ATS 0, patients with asthma, but not fulfilling the criteria of severe asthma refractory to treatment according to the ATS guidelines; ATS 1, suffering from asthma and fulfilling the criteria of severe asthma refractory to treatment according to the ATS guidelines; FEV1, forced expiratory volume in 1 sec; FVC, forced vital capacity; FEV1:FVC, ratio of FEV1 to FVC; r, linear correlation coefficient; r^2^, multiple correlation coefficient squared; ATS, American Thoracic Society; P, P-value.

**Table VII:** Haplotype-specific scores for depression using the haplotype effects model.

Genotype			Haplotype		
		
ER22/23EK	N363S	BclI	Frequency	Score	P-value
GG	GG	GG	0.05797	−0.88383	0.37679
GG	AA	CC	0.39565	−0.71309	0.47579
GG	GG	CC	0.02783	−0.24739	0.80461
AA	AA	GG	0.02235	0.36511	0.71503
AA	AA	CC	0.00725	0.63020	0.52856
GG	AA	GG	0.48776	0.97533	0.32940

**Table VIII:** Haplotype-specific scores for STAI-I using the haplotype effects model.

Genotype			Haplotype		
		
ER22/23EK	N363S	BclI	Frequency	Score	P-value
GG	AA	CC	0.39565	−0.63527	0.52525
GG	GG	GG	0.05797	−0.57593	0.56466
AA	AA	CC	0.00725	−0.29875	0.76513
AA	AA	GG	0.02235	−0.22619	0.82106
GG	GG	CC	0.02783	0.01604	0.98720
GG	AA	GG	0.48776	0.98623	0.32402

STAI-I, state-anxiety inventory.

**Table IX:** Haplotype-specific scores for STAI–II using the haplotype effects model.

Genotype			Haplotype		
		
ER22/23EK	N363S	BclI	Frequency	Score	P-value
GG	AA	CC	0.39565	−1.21509	0.22433
GG	GG	GG	0.05797	−1.0306	0.30273
AA	AA	GG	0.02235	−1.01922	0.30810
GG	GG	CC	0.02783	0.63846	0.52317
GG	AA	GG	0.48776	1.53739	0.12420
AA	AA	CC	0.00725	2.21614	0.02668

STAI–II, trait-anxiety inventory.

**Table X:** Haplotype-specific stores for breathlessness using the haplotype effects model.

Genotype			Haplotype		
		
ER22/23EK	N363S	BclI	Frequency	Score	P-value
GG	GG	GG	0.05998	−1.20047	0.22995
AA	AA	CC	0.00720	−0.89380	0.37143
AA	AA	GG	0.02255	−0.76096	0.44668
GG	AA	CC	0.39722	−0.67443	0.50004
GG	GG	CC	0.02741	0.92493	0.35500
GG	AA	GG	0.48451	1.33597	0.18156
